# “A Lot of People Can’t Do This”: Voices of Direct Care Workers in Assisted Living

**DOI:** 10.1093/geroni/igaf122.2140

**Published:** 2025-12-31

**Authors:** Candace Kemp, Jennifer Morgan

**Affiliations:** Georgia State University, Atlanta, Georgia, United States; Georgia State University, Atlanta, Georgia, United States

## Abstract

In this paper, our focus is on giving voice to and understanding the lived on-the-job experiences of assisted living (AL) staff, including direct care workers and engagement staff. We present analysis of qualitative interviews with staff (n = 77) collected as part of a study focused on meaningful engagement and quality of life for AL residents with dementia. We collected data in 8 diverse care communities located in and around Atlanta, Georgia, each studied for one year between 2019 and 2024. Staff voices unified around having “heart” for care work being drawn to and remining in the job for the “love of residents.” Most characterized their work as “hard,” requiring “patience,” and “not something just any can do.” Consequently, many viewed the material rewards and status of their work as being incommensurate with its importance and level of difficulty. One care worker explained, “I wish it pays more” because “wiping behinds and dealing with different personalities . . . I should be eating off of a silver platter, because a lot of people can’t do this.” Most reported ongoing job quality and workload challenges. Additional challenges arose from residents’ family members being uninvolved or well-intentioned yet lacking sufficient dementia knowledge. Empowerment practices that increase worker capacity and improve job quality and career mobility are needed across assisted living. The role of training, competency development, specifically in the area of dementia care, and employer of choice practices to support career development across this sector are discussed.

